# Supplementary material for: Does a learner-centered approach using teleconference improve medical students’ psychological safety and self-explanation in clinical reasoning conferences? a crossover study
Source: PLoS One. 2021 Jul 9;16(7):e0253884. doi: 10.1371/journal.pone.0253884 (PMC8270125; doi:10.1371/journal.pone.0253884)
Supplement: S1 Table — (PDF) [file pone.0253884.s001.pdf]

Supplement 1 Table. Facilitator characteristics

	Gender	Postgraduate year	Number of facilitation	Group in charge
Facilitator A	Male	5	1	Group 2
Facilitator B	Male	5	2	Group 1 and Group 2
Facilitator C	Male	7	1	Group2
Facilitator D	Male	12	2	Group 1 and Group 2
Facilitator E	Male	17	1	Group 1
