# Supplementary material for: Does a learner-centered approach using teleconference improve medical students’ psychological safety and self-explanation in clinical reasoning conferences? a crossover study
Source: PLoS One. 2021 Jul 9;16(7):e0253884. doi: 10.1371/journal.pone.0253884 (PMC8270125; doi:10.1371/journal.pone.0253884)
Supplement: S1 Fig — (PDF) [file pone.0253884.s003.pdf]

Supplement 1. Facilitator guide

Teleconference instructional design

Learning objectives of the conference:

1. To build knowledge that can be applied in a clinical setting and to develop clinical reasoning skills by bridging the knowledge gap.
2. To be motivated in learning and to build an effective self-study plan.
3. To be able to speak up actively by using learner-centered approach teleconference.

Facilitator's rolls

1. Use a "learner-centered" approach by interfering only when necessary.
2. Provide re-direction if necessary.
3. Encourage students to participate in group discussion when necessary.
4. Assist students by bridging the knowledge gap.
5. Facilitator is not allowed to appoint students at any time.

Flow of the conference

1. Introduction

-Introduce yourself and your roll during the conference.

-Explain that you are going to collect data by using questionnaire

2. During the conference

-Intervene to discussions only necessary

3. After the conference

-Ask students if they have any questions and give them feedback

-Ask students to answer questionnaire
