# Supplementary figures and images for: Does a learner-centered approach using teleconference improve medical students’ psychological safety and self-explanation in clinical reasoning conferences? a crossover study
Source: PLoS One. 2021 Jul 9;16(7):e0253884. doi: 10.1371/journal.pone.0253884 (PMC8270125; doi:10.1371/journal.pone.0253884)

Supplement 2. CONSORT

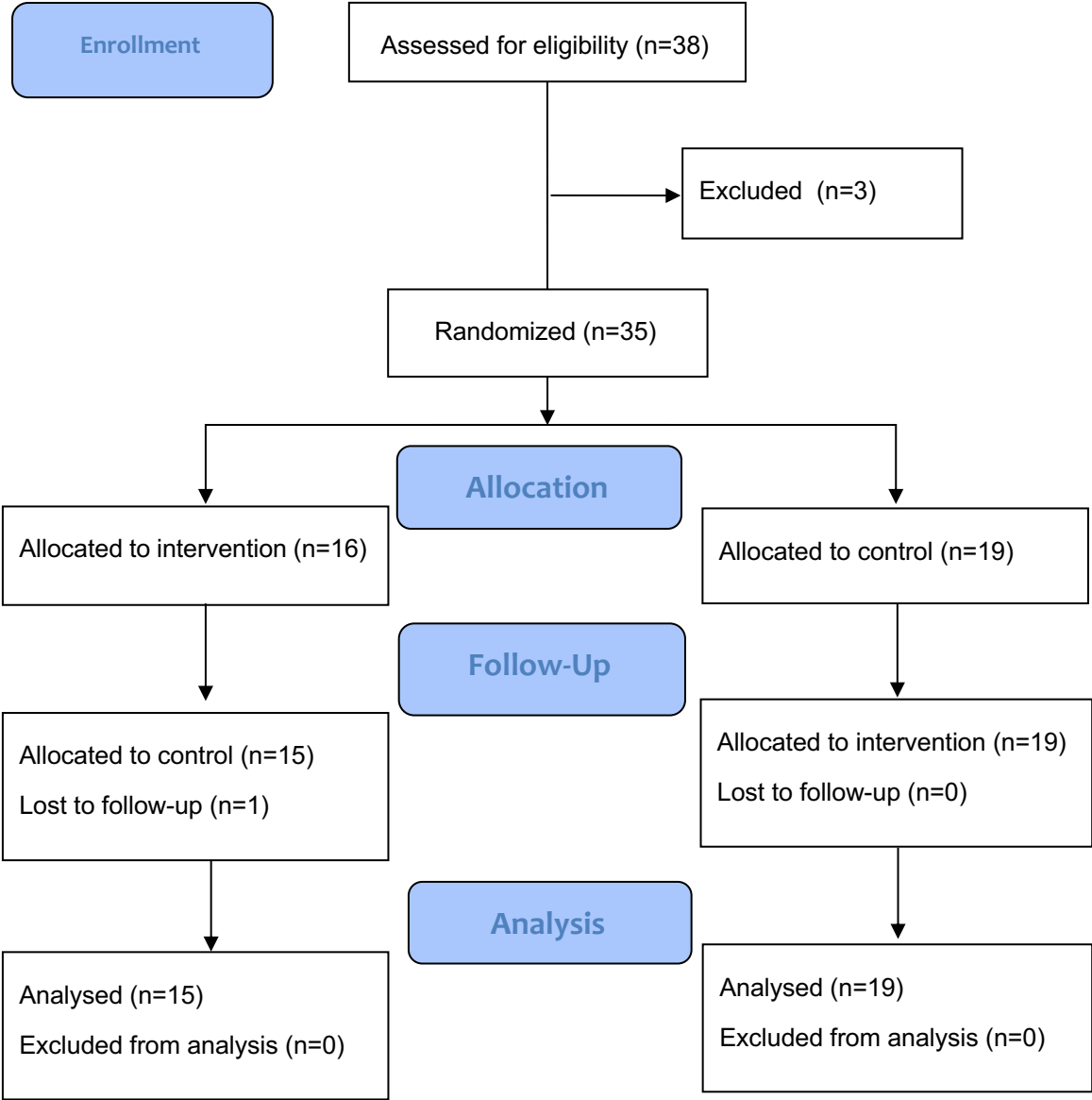

Supplement: S2 Fig — (PDF) [file pone.0253884.s004.pdf]
