# Supplementary material for: Does a learner-centered approach using teleconference improve medical students’ psychological safety and self-explanation in clinical reasoning conferences? a crossover study
Source: PLoS One. 2021 Jul 9;16(7):e0253884. doi: 10.1371/journal.pone.0253884 (PMC8270125; doi:10.1371/journal.pone.0253884)
Supplement: S3 Fig — Questionnaire used in the study (in Japanese). (PDF) [file pone.0253884.s005.pdf]

質問表:

1. Psychological Safety に関する質問.

(1: 全くそう思わない　7: 大いにそう思う)

1. チームの中で間違いをおかすと, たいてい非難される。(R)
2. チームのメンバーは, 課題や難しい問題を抽出することができる。
3. チームのメンバーは, 自分と異なることを理由に他者を拒絶することがある。(R)
4. このチームなら, 安心してリスクを負うことができる。
5. このチームのメンバーに対して, 助けを求めることは難しい。(R)
6. このチームには私の成果を意図的におとしめるような行動をとるものはいない。
7. チームのメンバーと仕事をする中で, 私個人の能力と才能は, 尊重され役に立っている。

2. 今回今回のカンファレンスについて回答してください.

(1: 全く思わない　4: どちらでもない　7: 大いにそう思う)

- 能動的に参加できたと思いますか?
- 満足しましたか?

3. 対面形式とサテライト形式ではどちらのカンファレンス形式が良いと思いますか?

1: 対面式　2: どちらとも言えない　3:サテライト形式
