# Supplementary material for: Does a learner-centered approach using teleconference improve medical students’ psychological safety and self-explanation in clinical reasoning conferences? a crossover study
Source: PLoS One. 2021 Jul 9;16(7):e0253884. doi: 10.1371/journal.pone.0253884 (PMC8270125; doi:10.1371/journal.pone.0253884)
Supplement: S4 Fig — Questionnaire used in the study (in English). (PDF) [file pone.0253884.s006.pdf]

Questionnaire:

1. Psychological Safety

(1= very inaccurate; 7= very accurate.)

1. If you make a mistake on this team, it is often held against you. (R)
2. Members of this team are able to bring up problems and tough issues.
3. Members of this team sometimes reject others for being different. (R)
4. It is safe to take a risk on this team.
5. It is difficult to ask other members of this team for help. (R)
6. No one on this team would deliberately act in a way that undermines my efforts.
7. Working with members of this team, my unique skills and talents are valued and utilized.

2. Please answer to each question for the conference.

(1. Strongly disagree 4. Neither agree nor disagree 7= Strongly agree)

- Were you able to participate to the conference actively?
- Are you satisfied with the conference?

3. Which types of conference do you prefer?

1 Traditional, live-style conference 2. Neutral 3. Learner-centered approach teleconference
