# Supplementary material for: Does a learner-centered approach using teleconference improve medical students’ psychological safety and self-explanation in clinical reasoning conferences? a crossover study
Source: PLoS One. 2021 Jul 9;16(7):e0253884. doi: 10.1371/journal.pone.0253884 (PMC8270125; doi:10.1371/journal.pone.0253884)
Supplement: Attachment — Submitted filename: Response to Reviewers.docx [file pone.0253884.s007.docx]

June 3, 2021

Dear Editors and Reviewers

**Title:** Does a learner-centered approach using teleconference improve medical students’ psychological safety and self-explanation in clinical reasoning conferences? A crossover study

**Reference number:** PONE-D-21-05048

Dear Editors and Reviewers,

Thank you for your e-mail on May 17, 2021, regarding our manuscript, ”***Does a learner-centered approach using teleconference improve medical students’ psychological safety and self-explanation in clinical reasoning conferences? A crossover study****,*” and for the valuable comments of the associated editor and referees. I have attached our marked-up copy of our manuscript, revised manuscript, as well as a point-by-point response to the associated editor and referees’ comments.

Responses to the comments by the academic editor and reviewers.

**Responses to the comments by the reviewer #2:**

*Q1. The abstract section, should be rewording depend on your results, Figure3 and Figure 2 not match, please check.*

*Q4. Page 11, Results section, should be rewording too, because “learner centered approach teleconference was preferred by fifteen students (44.1%), traditional conferences by eight students (23.5%), and neutral by eleven students (32.4%) (Fig 3). Twenty-nine (85.3%) students did self-explanation at least once in the learner-centered approach teleconference compared to ten students (29.4%) in the traditional live conference style (p<0.01).” but authors’ Figure 3 presented that teleconference (44.1%) and N=15 not match Figure 2. teleconference group and N=19.*

**Reply:**

We sincerely appreciate all your important comments. We reworded the abstract section and the result section as described below. First, we deleted line 44 and lines 49-51 on page 3 (on Revised manuscript with track changes, abstract section) to make the abstract concise and to focus more on psychological safety and the number of students who speak up during each conference.

Figure 3 was delated during the previous revision because it was pointed out that it doesn’t add to the paper over the text on lines 228-230 (page 14). N=15 and n=19 on Figure 2 is the number of students assigned to Group 1 and Group 2, respectively. Additionally, we added “n=34” next to the name of each conference in Figure 2 for readable purposes.

**Change:**

We delated line 44 and lines 49-51 on Page 3 (on Revised manuscript with track changes).

Figure 3 was delated during the previous revision.

“n=34” was added on Figure 2, next to the name of each conference.

*Q2. Page 11, line181-185, Results section, “…after the first conferences. The median age of the participants were 23 (21–28) years, and 25 students 184 (71.4%) were men. Two facilitators participated in learner-centered approach teleconference twice and 3 facilitators participated once...” for readable and contribution, suggest authors add descriptive statistics chart.*

**Reply:**

We appreciate your suggestion for adding a descriptive statistics chart. We added a new Table (Table 1) which describes the statistics chart of participants. Additionally, we added a new supplement (S1 Table) which describes the character of facilitators.

Statistical analysis for gender and age of students was added on line 204-205, Page 12.

Because we added a new Table 1, the previous Table 1 and Table 2 are now Table 2 and Table 3, respectively.

**Change:**

We added a new Table 1 and S1 Table.

We added line 209-211 (page 13)

“Gender and age were analyzed using Fisher’s t-test and the Mann-Whitney U-test, respectively.”

Previous Tables 1, 2, 3, and 4 are now Tables 2, 3, 4, and 5, respectively.

*Q3. For easy read, suggest authors add participants sample size in the Table 1. and Table 2.*

**Reply**:

Thank you very much for your excellent suggestion. We added sample size in Table 1 and Table 2. Also, we added sample size on new Table 3 and Table 4.

Because we added a new Table 1, which is the participant characteristic, the previous Tables 1, 2, 3, 4 are now Table 2, 3, 4, and 5, respectively.

**Changes:**

We added sample size in new Table 2, Table 3, Table 4, and Table 5.

*Q5. The discussion and conclusions section should follow the Results section.*

**Reply:**

Thank you very much for your invaluable comments. During the previous revision, we added the subanalysis and had reworded the discussion and conclusion sections based on the results and limitations. In this revision, we have reworded additional changes so the discussion and conclusion follow the result section.

**Change**:

Lines 321-323 (Page 21)

Add “In addition, students in the teleconference could receive direct feedback from experienced facilitator, which may contribute to their high satisfaction and preference.”

Line 395-397 (Page 26) on revised manuscript with track changes.

Delated “enabling educators to assess their understanding levels more effectively.”

Additional changes are shown in red color on revised manuscript with track changes.

*Q6. It is suggested that authors have the paper proofread by a professional proofreader who is a native English speaker.*

**Reply:**

We sincerely appreciate all your important comments. We submitted our manuscript to Editage ([www.editage.com](http://www.editage.com)), which is a native English speaker for proofreading. All changes are highlighted in red on the marked-up copy of our manuscript.

For “Financial disclosure,” the authors received no specific funding for this work.

We hope that the revised manuscript contains suitable responses to the comments, and we think that it has been significantly improved over the initial submission. We trust that our manuscript is now suitable for publication in *PLOS ONE*.

Thank you in advance for your kind consideration of our work.

Sincerely yours,

Kiyoshi Shikino, MD, PhD, MHPE

Department of General Medicine, Chiba University Hospital

1-8-1, Inohana, Chuo-ku, Chiba-city, Chiba, Japan

Tel. +81-43-222-7171 (Ext. 6438); +81-43-224-4758 (Direct line)

Fax. +81-43-224-4758

E-Mail: kshikino@gmail.com
